# “I don't know why I am in hospital”: amnesia in non-fatal hanging

**DOI:** 10.1016/j.heliyon.2021.e08365

**Published:** 2021-11-11

**Authors:** S.M. Yasir Arafat, A.K.M. Bazlul Karim

**Affiliations:** a Department of Psychiatry, Enam Medical College and Hospital, Dhaka, 1340, Bangladesh; b Department of Neurosurgery, Enam Medical College and Hospital, Dhaka, 1340, Bangladesh

**Keywords:** Non-fatal hanging, Amnesia, Retrograde amnesia, Anterograde amnesia, Suicide in Bangladesh

## Abstract

There is an extreme dearth of empirical studies assessing the neuropsychiatric outcome of non-fatal hanging that indicates little attention to the area has been paid. We aimed to report the memory disturbances of 14 cases after an immediate non-fatal hanging attempt. We conducted the study from August 2020 to June 2021 and collected data from 14 hospitalized patients after an immediate non-fatal hanging attempt. We conducted series of clinical examinations to assess the memory disturbance and recorded it. Among the 14 cases, four (28.8%) were males and the rest ten (71.2%) were females. The mean age of the cases was 25.28 ± 8.19 years ranging from 15-40 years. All the cases had retrograde amnesia while six (42.9%) had both anterograde and retrograde amnesia. The current pilot study reported the distribution of memory disturbances among fourteen cases of non-fatal hanging that adds preliminary findings to the under-researched area and warrants further empirical studies to generalize the study results.

## 1 Introduction

Suicide is a global public health problem (Orsolini et al., 2020). About 7,00,000 people are dying every year by suicide (World Health Organization, 2021). Many years of active life have been lost due to suicide. It also causes a significant psychological burden to the family members. It is an outcome of an extremely complex interaction between genetics, neurobiology, environment, psychology, religious belief, cultural influence, psychiatric disorders (Orsolini et al., 2020; De Berardis et al., 2018). Therefore, identification of any risk factors with specific relations is challenging. However, the non-fatal attempt is an important risk factor for suicide (Ostertag et al., 2019). Newer studies are coming out to identify the neurobiological markers of suicide as well as to explain the behavior precisely (Orsolini et al., 2020; De Berardis et al., 2018).

Hanging is one of the lethal methods of suicide preferably used across the globe with a different mechanism leading to death (Aneja et al., 2017). The outcome of hanging widely varies such as death, various forms of permanent anoxic brain injury, and even complete recovery (Jawaid et al., 2017). Various forms of neuropsychiatric presentation have been noted in hypoxic brain injuries such as Korsakoff's psychosis, memory disturbances, posterior reversible encephalopathy syndrome, neurological deficits, mental disorders, personality change, and impairment of mental functions (Aneja et al., 2017; Medalia et al., 1991; Jawaid et al., 2017; Zabel et al., 2005). Among the neurocognitive changes, memory disturbances in the form of both retrograde and anterograde amnesia have been reported (Andriuskeviciute et al., 2016; Aneja et al., 2017; Medalia et al., 1991; Jawaid et al., 2017; Zabel et al., 2005). However, studies on the neurological sequelae of non-fatal hanging are restricted to retrospective observation and case reports. There is an extreme dearth of empirical studies assessing the neuropsychiatric outcome and its associated risk and prognostic factors in non-fatal hanging indicating that little attention to the area (Aneja et al., 2017; Medalia et al., 1991). Against this background, here we report the memory disturbances of 14 cases after an immediate non-fatal hanging attempt. We aimed to assess whether there is any memory disturbance among the patients after a non-fatal hanging. Additionally, we intended to see the rate and type of memory disturbances. This study would add an important contribution to the field with scarce evidence and act as a baseline pilot study describing the distribution of memory problems.

## 2 Method

We conducted the study from August 2020 to June 2021 and collected data from 14 hospitalized patients after an immediate non-fatal hanging attempt. We intended to include all the consecutive patients admitted after a non-fatal hanging attempt. We excluded the patients who refused to stay in the psychiatry ward for further assessment. The was conducted in a private hospital setting where expenses are being incurred from out of the pocket of the patients which is an important determinant for refusing a longer hospital stay. After an attempt, the patients were presented to the emergency department, from where they were admitted at neuro-intensive care unit (ICU) under the department of neurosurgery department. After an immediate stabilization in neuro ICU, they were transferred to the department of psychiatry for the mental state examination (MSE) and further management. The first author (psychiatrist) conducted a series of MSEs to identify the potential risk factors, psychiatric comorbidity, and memory disturbances. During the ICU stay, necessary investigations including neuroimaging (magnetic resonance imaging (MRI) of brain and cervical spine) were performed to identify the hypoxic-ischemic injury in the brain and mechanical injury in cervical vertebra. We used Microsoft Excel (2010) software to store and analyze the data. Simple descriptive analysis was performed and expressed as frequency, percentages, mean, and standard deviation. This study is a part of our project whose ethical approval was taken from the ethical review committee of Enam Medical College on August 08, 2020 (EMC/ERC/2020/08-1). Informed written consent was obtained from the admitted patients and/or legal guardians whichever was appropriate, without any influence.

## 3 Results

Among the 14 cases, four (28.8%) were males and the rest ten (71.2%) were females. The mean age of the cases was 25.28 ± 8.19 years ranging from 15-40 years. All the cases had retrograde amnesia while six (42.9%) had both anterograde and retrograde amnesia. Some differences have been presented in Table 1. The differences were not statistically significant and none of the cases had any mentionable changes in MRI of the brain and cervical spine.

**Table 1:** Amnesia among non-fatal hanging (n = 14).

Variable	Retrograde amnesia	Anterograde and retrograde amnesia	Total [n (%)]
Age	27.37 ± 8.45 (18–40)	22.5 ± 7.63 (15–35)	25.28 ± 8.19 (15–40)
Sex			
Male	1 (25)	3 (75)	4 (100)
Female	7 (70)	3 (30)	10 (100)
Total	8 (57.1)	6 (42.9)	14 (100)

## 4 Discussion

Proportion, distribution, and type of memory disturbances after a non-fatal hanging have been a poorly researched area with sparse and old literature. Here, we report the distribution of amnesia in 14 cases, those admitted into hospital after an immediate non-fatal suicidal attempt by hanging. We found that all of the reported cases had retrograde amnesia with the inability to remember the event of the suicidal attempt and about 44% (n = 6) had additional memory problems where none of the cases had identifiable changes in MRI of the brain. The finding indicates that memory disturbances could an unavoidable consequence in non-fatal hanging. The mean age of the cases was about 25 years and about 70% of the cases were females. The age and sex distribution of the cases support the usual pattern of suicide in the country (Arafat, 2019). However, the distribution of memory disturbances was different in rate when compared to the other studies. Previously, we reported three cases with memory disturbances (Arafat et al., 2020). All three cases had retrograde amnesia albeit two were suicidal and one was accidental hanging with brain edema in both frontal lobe and hemorrhage in both thalami (Arafat et al., 2020). Interestingly, one of the cases had hysterical retrograde amnesia. The most identified robust study assessing the neurological outcomes of 101 cases from India found 42% (n = 39) had retrograde amnesia which is quite different from our study (Jawaid et al., 2017). The possible explanations could be the different sample where our patients could be more severe as all the cases had initial ICU management before transferring to the psychiatry department. Another case reported found organic amnestic syndrome in a 22 years old boy after a non-fatal hanging (Aneja et al., 2017). Although we reported a case with hysterical amnesia previously in this current study we didn't find any such cases. It is important to note that the duration of stay in the psychiatry department was 2–3 days only which may preclude the disclosure. However, previous studies reported hysterical amnesia and repeated authors raised the issue to be explored adequately (Arafat et al., 2020; Berlyne and Strachan, 1968). Medalia et al. (1991) reported two cases with isolated memory deficits (anterograde and retrograde) after non-fatal suicidal hanging. Zabel et al. (2005) assessed two adolescents with non-fatal attempts comprehensively and reported amnesia with recovery in one survivor. Another incident of recovery of initially lost memory with treatment was reported in a case from India after a hypoxic-ischemic encephalopathy due to hanging (Changal et al., 2013).

Previous reports identified amnesia in non-fatal hanging without any identifiable changes in the brain measured by neuroimaging (CT scan and MRI) (Arafat et al., 2020; Medalia et al., 1991). Cerebral ischemia, edema, hypoxia, anoxia have been thought to the attributing factor for this amnesia (Jawaid et al., 2017; Aneja et al., 2017; Medalia et al., 1991; Berlyne and Strachan, 1968). However, hysterical amnesia is also a potential presentation, even though the frequency could be lower. Psychological sufferings that herald to the suicidal attempt mount more when rescued from the attempt due to the stigma towards suicidal behavior that resulting in shame and hindrance of the potential risk factors. Certainly, further robust studies are warranted to look into the pathophysiology of amnesia after hanging.

The study has several limitations. Firstly, we couldn't follow up with the patients which limit the results to identify the course of the amnesia. Secondly, we collected the cases of those admitted to the hospital through the emergency department. Therefore, results may not be generalized. Thirdly, we collected the cases from a single private tertiary care hospital that limits the generalization of the study results. Fourthly, the number of cases was relatively small that limit the generalization of the study results. Fifthly, the memory disturbance was assessed by the clinical interview that raises the issue of objectivity of the results. We didn't assess the other domains of cognition and risk factors for this amnesia. Sixthly, we couldn't control the psycho-social variable even though the clinical examination and investigations revealed nothing contributory to the amnestic features.

## 5 Conclusions

The current pilot study reported that memory disturbances could be an unavoidable consequence in non-fatal suicide as all the patients of this study had memory disturbances. The findings on this study add preliminary contribution to the under-researched area and warrants further empirical studies to generalize the study results.

## Declarations

### Author contribution statement

All authors listed have significantly contributed to the investigation, development and writing of this article.

### Funding statement

This research did not receive any specific grant from funding agencies in the public, commercial, or not-for-profit sectors.

### Data availability statement

Data will be made available on request.

### Declaration of interests statement

The authors declare no conflict of interest.

### Additional information

No additional information is available for this paper.
